# Imputation of adverse drug reactions: Causality assessment in hospitals

**DOI:** 10.1371/journal.pone.0171470

**Published:** 2017-02-06

**Authors:** Fabiana Rossi Varallo, Cleopatra S. Planeta, Maria Teresa Herdeiro, Patricia de Carvalho Mastroianni

**Affiliations:** 1 São Paulo State University (UNESP), School of Pharmaceutical Sciences, Araraquara, São Paulo, Brazil; 2 CAPES Foundation, Ministry of Education of Brazil, Brasília—DF, Brazil; 3 Departamento de Ciências Médicas—Universidade de Aveiro, Aveiro, Portugal; Kinki Daigaku, JAPAN

## Abstract

**Background & objectives:**

Different algorithms have been developed to standardize the causality assessment of adverse drug reactions (ADR). Although most share common characteristics, the results of the causality assessment are variable depending on the algorithm used. Therefore, using 10 different algorithms, the study aimed to compare inter-rater and multi-rater agreement for ADR causality assessment and identify the most consistent to hospitals.

**Methods:**

Using ten causality algorithms, four judges independently assessed the first 44 cases of ADRs reported during the first year of implementation of a risk management service in a medium complexity hospital in the state of Sao Paulo (Brazil). Owing to variations in the terminology used for causality, the equivalent imputation terms were grouped into four categories: definite, probable, possible and unlikely. Inter-rater and multi-rater agreement analysis was performed by calculating the Cohen´s and Light´s kappa coefficients, respectively.

**Results:**

None of the algorithms showed 100% reproducibility in the causal imputation. Fair inter-rater and multi-rater agreement was found. Emanuele (1984) and WHO-UMC (2010) algorithms showed a fair rate of agreement between the judges (k = 0.36).

**Interpretation & conclusions:**

Although the ADR causality assessment algorithms were poorly reproducible, our data suggest that WHO-UMC algorithm is the most consistent for imputation in hospitals, since it allows evaluating the quality of the report. However, to improve the ability of assessing the causality using algorithms, it is necessary to include criteria for the evaluation of drug-related problems, which may be related to confounding variables that underestimate the causal association.

## 1 Introduction

The adverse drug reaction (ADR) causality assessment is a routine procedure in Pharmacovigilance[1], because it allows assessing drug safety parameters and the relationship and likelihood between drug exposure and the occurrence of ADR of health technologies in the post-marketing period.

Since the 1970s, different methods to standardize the evaluation of the causal association of ADRs have been available, ranging from small questionnaires to comprehensive algorithms[2].

The development of these tools, which are ordinary to use[3] and require minimal expertise to be employed[1,4], aims to solve methodological bias, reliability, and validity issues in the imputation of drug-induced adverse effects[5]. However, the main advantage arises from the possibility of decentralizing the causality assessment from the medical diagnosis, extending it to different health care levels: academics, the pharmaceutical industry, and health agencies[6].

By standardizing ADR causality assessment, the uncertainty of the association between a drug and an adverse event will not be reduced, but semi-quantitatively categorized[2] in different links of probability.

Establishing a causal link may influence the rationale for the correlation of an event that occurs to drug consumers[7]; therefore, the results of the causality assessments using algorithms must be reproducible. This is important to ratify the viability of their employment in pharmacovigilance[8], as well as their capacity to detect ADR signals[9,10]. This is because the higher the agreement on a defined ADR causal link, the more robust the hypothesis about the relationship between the use of a medication and the adverse event observed, allowing the communication of the risk and, therefore, the implementation of risk minimization and patient safety plans.

Because serious ADRs lead to hospitalization, it is necessary to assess the causality in the tertiary health care level. However, there are few data about the agreement on ADR causality assessment using different algorithms in patients hospitalized in internal medicine units in developing countries.

It is known that most ADR evidence arises from hospitals, due to the high risks associated with treatments in the tertiary health care level[11]. Therefore, the causality assessment in high complexity institutions contributes to: i) the early recognition of adverse effects, which helps to prevent iatrogenic complications; ii) therapy optimization[2]; iii) establishing barriers to prevent recurrence; iv) reducing the time of hospitalization and unnecessary burden with hospitalizations that could be avoided[12].

This study aimed to compare the results of the imputation of ADRs using different algorithms in a Brazilian public hospital, to identify the most appropriate for establishing causal associations between medication use and the occurrence of adverse events.

## 2 Material and methods

### 2.1 Study design

We assessed the causality of all of the ADRs reported by health professionals during the first year of implementation of the pharmacovigilance service (March 2012 until March 2013) in a general assistance, public, medium complexity (secondary health care level) hospital with 104 beds located in the state of São Paulo.

### 2.2 Selection of algorithms

Twenty-nine (29) algorithms for ADR causality assessment were identified by literature review. Nineteen (19) were excluded for the following reasons: absence of equivalent terminology for the level of imputation of ADRs (n = 6); inclusion of information that is not required for the causality assessment in Brazil (n = 3); tools that were developed for the assessment of specific ADRs (n = 3); and no access to the article (n = 7).

The ten algorithms (Table 1) considered eligible for the study included the combination of five main criteria for the causality assessment[13], namely: i) plausible temporality; ii) prior bibliographic description of the adverse effects related to the use of the drug involved; iii) alternative causes; iv) positive withdrawal (discontinuation of the drug with improvement of the ADR); v) positive rechallenge (reintroduction of the drug with reappearance of the ADR).

**Table 1:** Algorithms selected for the causality assessment of adverse drug reactions in a public and general hospital in the State of Sao Paulo, Brazil (n = 10).

Algorithm	Reference (year)
Karch and Lasagna	Clin Pharmacol Ther (1977); 21: 247–254
Blanc *et al*.	Clin Pharmacol Ther(1979); 25: 493–498
Kramer *et al*.	JAMA(1979); 242: 623–632.
Naranjo *et al*.	Clin Pharmacol Ther (1981); 30: 239–245
Jones	Fam Community Health (1982); 5: 58–67
Emanuelli	Drug Inf J (1984); 18: 303–306
Mashford	Drug Inf J (1984); 18: 271–273
Venulet *et al*.	Int J Clin Pharmacol Ther (1986); 24: 559–568
WHO-UMC	Available from: http://who-umc.org/Graphics/24734.pdf
Gallagher *et al*.	PLoS One (2011), 6 (12): e28096

The use of the WHO-UMC system for standardized case causality assessment. World Health Organization (WHO)—Uppsala Monitoring Centre. [Last accessed on 2013 Apr 10]. Available from: http://www.who-umc.org/Graphics/24734.pdf.

Owing to the quantitative and qualitative variability in the terminologies used to express the results of the imputation of ADRs in the included algorithms, the nomenclature developed by Macedo et al. (2005)[13] was used. To improve the accuracy of the comparison, the equivalent terms of the likelihood level were grouped into four major categories: definite, probable, possible and unlikely.

### 2.3 Causality assessment

Using 10 causality algorithms (Table 1), four judges: FRV (rater A), ADFS (rater B) SPS (rater C) and IO (rater D) independently assessed the first 44 cases of ADRs reported to the hospital’s risk management service during its first year of implementation.

The group of judges who conducted the analysis included: a clinical pharmacist of the hospital (rater A) who had PhD in Pharmaceutical Sciences and 8 years of professional experience with pharmacovigilance issues; three pharmacy undergraduate students (raters B, C and D) who were in the last year of the course and had previously experience in pharmacovigilance´s scientific research for at least 1 year. The students were trained, in order to standardize the analysis of causal association. The 12-hour training included: 1) discussion of scientific papers on the subject (evaluation of ADR causality; evaluation of ADR causality with different decision algorithms, application of Austin Bradford-Hill’s criteria in pharmacoepidemiological studies); 2) directed study (comparison and critical analysis) of the algorithms used; 3) simulation of an ADR causality assessment with a fictional case[14].

The cases of ADRs reported and selected for the study contained at least the following information: i) suspected drug (start and end date); ii) a brief description of the event (start and end date, data of laboratory tests when relevant); iii) polypharmacy (start and end date); iv) the patient’s medical history; v) relevant interventions.

We considered ADR any noxious, unintended, or undesired effect of a drug occurring at doses used in humans for prophylaxis, diagnosis, or therapy[15].

The clinical manifestations reported were classified according to seriousness and expectance. Serious ADR were defined as those causing hospitalization, those that were fatal or life-threatening, or those that resulted in significant changes in patient treatment (thereby prolonging hospitalization)[16].

Informational drug sheets approved by the National Agency of Sanitary Surveillance (ANVISA) and monographs, such as those in the DRUGDEX (MICROMEDEX®database), Uptodate® database and LexiComp Manole (2009) were consulted to verify the expectancy of ADR.

The results of imputation obtained with the ten algorithms were compared to analyze the agreement between the judges and the feasibility of the algorithms in the causality assessment in hospitals.

### 2.4 Statistical analysis

Two descriptive statistics were used to measure the nominal agreement between two or more raters: Cohen´s kappa and Light´s kappa.

Cohen´s kappa measure the degree of concordance between two judges. The analysis carried out by FRV (rater A) was considered gold-standard to calculate the inter-rater agreement between judges B, C and D.

Light´s kappa is a multi-rater statistic which measures the degree of concordance among multiple judges without gold-standard. It is an extension of Cohen’s kappa. For both tests, we considered α = 0.05, 95%CI for all analyses. Values were interpreted according to Landis and Koch protocol (1977)[17] (Table 2).

**Table 2:** Interpretation of the Kappa (K) value.

Kappa value	Interpretation of the agreement
<0.00	Less than chance
0.00 ǀ– 0.21	Slight
0.21 ǀ– 0.41	Fair
0.41 ǀ– 0.61	Moderate
0.61ǀ–0.81	Substantial
0.81ǀ–ǀ1.00	Almost perfect

### 2.5 Research ethics committee

This study (E-015/10 protocol) was approved by the Research Ethics Committee of the Instituto Lauro de Souza Lima.

## 3 Results

During the period of data collection, the risk management department received 24 ADRs reports that enclose 36 different types of clinical manifestations resulting from 19 drugs (Table 3). Owing to the causality imputation was carried out case to case, each judge independently assess 44 cases, since a single report may describe more than one clinical manifestation associated with only one drug or may signalize more than one suspected drug for the occurrence of a single clinical manifestation.

**Table 3:** Characteristics of the adverse drug reactions (ADR) analyzed, according to characteristic of patients, expectative, frequency, seriousness of the event (n = 44).

Characteristic of patient (age, sex and diagnosis)	ADR	Suspected Drug (s)	Expectative	Frequency^1^	Seriousness
42 years old, female, human immunodeficiency virus	Chemical phlebitis	azithromycin	Expected	1.0–10.0%	Non-serious
30 years old, male, viral meningitis	skin rash	[Ceftriaxone], [ketoprofen], [aciclovir]	Expected	[1.0%], [9.0%], [>3.0%]	Non-serious
32 years old, male, transverse loop colostomy	Shin rash	[Ciprofloxacin], [clindamycin]	Expected	>1,8%	Non-serious
51 years old, female, chronic obstructive lung disease	Tachycardia1, dyspnea2 and anxiety disorders3	[Ipratropium],[Fenoterol]	Expected1,2*	[Tachycardia <1.0%] [dyspnea 10.0%]	Serious
37 years old, male, chronic osteomyelitis	Chemical phlebitis	meropenem	Expected	2.0%	Non-serious
52 years old, female, hydronephrosis with ureteropelvic junction obstruction	Tachycardia1, malaise2 and vaginal burning sensation3	azithromycin	Expected1,2*	<1.0%	Non-serious
76 years old, female, dementia with Lewy bodies	Skin rash	carbamazepine	Expected	7.0%	Non-serious
37 years old, female, human immunodeficiency virus	Malaise1, tachycardia2, hyperhidrosis3	metoclopramide	Expected2*	Not defined2	Non-serious
65 years old, male, pleural effusion	Chemical phlebitis	meropenem	Expected	2.0%	Non-serious
68 years old, male, stroke	Shin rash1 and urticarial2	acetylsalicylic acid	Expected1,2	Not defined1,2	Serious
33 years old, male, human immunodeficiency virus	Red men syndrome	vancomycin	Expected	> 10.0%	Non-serious
84 years old, male, pneumonia	Skin rash	piperacillin and enzyme inhibitor	Expected	4.0%	Non-serious
84 years old, male, urinary tract infection	Pruritus	polymyxin B	Expected	Not defined	Non-serious
40 years old, male, Wernicke encephalopathy	Severe hypokalemia	polymyxin B	Expected	Not defined	Serious
42 years old, female, obesity, cellulitis	Irritation on the local of application	heparin	Expected	Not defined	Non-serious
49 years old, male, pulmonary tuberculosis	Skin rash	[Ceftriaxone], [clindamycin], [metronidazole]	Expected	[2.0%], [not defined], [not defined]	Non-serious
62 years old, female, chronic pressure ulcer	Nausea and vomit	dipyrone	Expected	Not defined	Non-serious
62 years old, female, chronic pressure ulcer	Acute kidney impairment	vancomycin	Expected	5.0%	Serious
35 years old, male, human immunodeficiency virus	Skin rash	piperacillin and enzyme inhibitor	Expected	4.0%	Non-serious
50 years old, female, chronic obstructive lung disease	Pain in the local of application1, tachycardia2	clarithromycin	Expected	≥0.1% and < 10.0%1, <1.0% 2	Non-serious
42 years old, female, cellulitis	Erythema	levofloxacin	Expected	< 1.0%	Non-serious
24 years old, male, human immunodeficiency virus	Anemia	zidovudine	Expected	< 1.0%	Serious
66 years old, female, chronic pressure ulcer	Mental confusion1, drowsiness2, constipation3, vomit4	morfine	Expected1-4	6.0%1, > 9.0%2,3, >10.0%4	Serious
74 years old, male, cellulitis	Myoclonus	morfine	Expected	< 1.0%	Serious

^1^Reference: Micromedex® database, Up to Date® database and Lexi-comp Manole (2009).

*Except to anxiety disorders, vaginal burning sensation, malaise and hyperhidrosis.

According to seriousness, seven ADR reports showed symptomatology classified as serious, since 4 of them prolonged hospital length-stay, 2 resulted in temporary disability and 1 was related to hospital admission.

After causality assessment, none of the algorithms showed 100% agreement between judges on the imputation of ADRs. Fair agreement was observed for both statistic tests (Cohen´s and Light´s kappa) (Table 4). Findings suggest the poor reproducibility of the algorithms in performing ADR imputation with different judges.

**Table 4:** Inter-rater and multi-rater agreement in adverse drug causality assessment, according to the statistical analysis with Cohen´s and Light´s kappa.

Algorithm	Cohen's Kappa			Light's Kappa
	Judge B	Judge C	Judge D	Multiple judges
	Kappa; IC (95%)	Kappa; IC (95%)	Kappa; IC (95%)	I Kappa; IC (95%)
Kart e Lasagna (1977)	0.14 (0.00–0.37)	036 (0.14–0.57)	0.29 (0.05–0.51)	0.23 (0.12–0.37)
Kramer *et al*. (1979)	0.21 (0.02–0.44)	0.53 (0.32–0.73)	0.34 (0.16–0.54)	0.19 (0.09–0.34)
Blanc *et al*. (1979)	0.16 (0.00–0.36)	0.37 (0.15–0.57)	0.49 (0.29–0.67)	0.29 (0.19–0.42)
Naranjo *et al*. (1981)	0.29 (0.03–0.55)	0.39 (0.13–0.65)	0.41 (0.16–0.69)	0.20 (0.08–0.39)
Jones (1982)	0.54 (0.25–0.76)	0.34 (0.03–0.59)	0.09 (0.00–0.37)	0.27 (0.12–0.46)
Emanuelli *et al*. (1984)	0.44 (0.19–0.67)	0.29 (0.04–0.54)	0.40 (0.16–0.64)	0.36 (0.21–0.53)
Mashford (1984)	0.26 (0.00–0.59)	0.53 (0.19–0.79)	0.54 (0.29–0.76)	0.33 (0.21–0.50)
Venulet *et al*. (1986)	0.16 (0.06–0.31)	0.32 (0.09–0.57)	0.37 (0.13–0.60)	0.15 (0.05–0.28)
WHO-UMC (2010)	0.32 (0.09–0.58)	0.37 (0.15–0.58)	0.41 (0.17–0.64)	0.36 (0.10–0.53)
Gallagher *et al*. (2011)	0.21 (0.01–0.42)	0.41 (0.21–0.60)	0.26 (0.04–0.50)	0.29 (0.17–0.43)
**Mean ± standard deviation**	**0.27 ± 0.13**	**0.39 ± 0.08**	**0.36 ± 0.12**	**0.27 ± 0.07**

The lower inter-rater agreement was observed for the judge B, except for Emanueli (1984), Jones (1986) and WHO-UMC (2010) algorithms (Table 4).

Venulet (k = 0.15), Kramer (k = 0.19), and Naranjo (k = 0.20) algorithms showed the worst multi-raters coefficient, indicating slight agreement on causal association. Moreover, the agreement was stronger in Emanueli (k = 0.36), Mashford (1984) and WHO-UMC (k = 0.36) algorithms, but not better than fair (Table 4).

## 4 Discussion

Our data suggest that WHO-UMC algorithm is the most consistent for causal imputation of hospital ADR that affected patients admitted to an internal medicine unit of a medium complexity hospital. The advantage of this tool is the semi-quantitative assessment of the causal likelihood and of the quality of the report; it has been used as a gold standard in causality studies[8,13]. Moreover, this tool was developed to evaluate the occurrence of adverse effects during the post-marketing period, which helps to achieve higher probability scores of causal association and a better reproducibility between the judges.

Emanueli (1984) algorithm, which contains a minimalist, simplified, dichotomous structure that considers only the clinical condition of the patient with alternative cause, may overestimate the cases of ADR and generate false-positive signals in risk communication, which is why it is not the most recommended for causality assessment in the context of this study.

For the remaining algorithms, we noted a weak agreement between the judges on ADR causality. Studies have shown a great variability in the results of imputation of ADR using different algorithms[8,13,18–22]. According to Shakir and Layton (2002)[10], the tools are inconsistent and sometimes of poor quality for signal detection. Furthermore, they have significant limitations that reduce the accuracy and reliability of the assessment of the probability of ADR[1].

Considering Naranjo et al. (1981) algorithm, data from previous study showed slight agreement between the judges[21], because it was developed and validated for the assessment of ADRs that occur during randomized clinical trials[19]. Other authors suggest the use of this tool for the imputation of ADR[21] due to its rapid implementation. However, we disagree this is the only factor to consider when choosing an algorithm. In addition to this aspect, the reliability of the results and the limitations of each tool, especially in the context of medication use (clinical trial *versus* post-marketing surveillance), should be considered. According to the data from our study, WHO-UMC (2010) partially meets these criteria.

We understand that it meets in an incomplete manner, because all of the analyzed algorithms do not include other factors that may be associated with adverse events, such as medication errors, product quality deviations, and suspected therapeutic ineffectiveness. Most consider in the assessment only the drug safety issues and neglect (Emanueli 1884; Blanc et al., 1979; Gallagher et al., 2011) or ambiguously (Karch Lasagna, 1977), subjectively (Naranjo et al., 1981; Mashford, 1984; WHO-UMC, 2010) or complexly (Kramer et al., 1979; Venulet et al., 1986) manage other factors that may be associated with adverse events. Even Gallagher et al. (2011) algorithm which was developed after the new definition of pharmacovigilance in 2002 did not include relevant information in the assessment, allowing underestimation of a causal association. This may also be correlated with the low agreement between the judges.

The arbitrary weighting given to the evaluation criteria is another limitation that may contribute to the inconsistency of algorithms[5,23]. This adds subjectivity inherent to the algorithm structure according to criteria these authors deem most important and give greater weighting in scoring. The causality assessment itself also includes some subjectivity[2,6]. Both situations described may contribute to the poor agreement between the algorithms in the imputation of the causal link of ADR.

Another evidence that may decrease the accuracy of risk communication is the absence of ADR reports of good quality and underreporting[10,24,25]. Poor or missing information in the reports makes it difficult assessing causality in details, differentiating between probable and possible cases[2], and finding a definitive causal association. Consequently, the assumptions are not robust enough to generate signals in pharmacovigilance, impairing the assessment of drug safety in the post-marketing period.

Nowadays, there is no gold-standard algorithm for the assessment of events occurring in primary care[6,26] and studies that compared the imputation of ADR reported to national pharmacovigilance centers[13,23]. At the tertiary health care level, Kane-Gill et al. (2012)[20] found strong agreement when comparing three algorithms by active search of retrospective cases of ADR in the intensive care unit. This can be explained by the ward where the study was conducted, the methodology (one judge) and the active search method. Critical patients are constantly monitored, so the records in medical charts are more complete, which allows the collection of better information and increases the robustness of causality assessment. However, the disadvantage of the active search is the time necessary to review medical records[27], which turns the process unfeasible.

Considering the limitations described, there is evidence that it is necessary to develop better quality tools that improve the diagnosis of ADR[26]. A strategy to increase the generation of pharmacovigilance signals is the monitoring of adverse drug events[28], and its evaluation criteria should be included in the algorithms to improve the reproducibility in the causal imputation. These criteria involve the assessment of any drug-related problem.

Therefore, in an attempt to minimize the described flaws and confounding variables during the assessment, the need, effectiveness, adherence and safety parameters should also be included in the algorithms in order to update the assessment with the new concepts of WHO (2002)[29] about post-marketing studies.

Considering drug use[28,30] and the intentional non-compliance with pharmacotherapy related to the diagnosis stereotypical diseases[31] are associated with undesirable effects, it is also necessary to evaluate the impact of ADRs on the patient. This would help to know the priorities, difficulties and factors that may motivate the use or discontinuation of therapy and therefore the occurrence of undesirable effects derived from these perceptions and practices.

Finally, although the literature presents a wide range of methods for the causality assessment, including computational approaches, algorithms are still viable alternatives to the causality assessment in hospitals, since these tools are easy to use, require little financial resources to be applied in the clinical routine and need minimal expertise to be applied[18]. Thus, it is important to update these algorithms in accordance with the new definition of pharmacovigilance, allowing the monitoring of adverse drug events[26], in order to minimize confounding variables associated with the causal imputation process and therefore improve the risk/benefit assessment of medications available on the market.

## 5 Conclusion

Our data show slight agreement on the ADR causality assessment for the majority of the tested algorithms. However, WHO-UMC (2010) algorithm showed fair reproducibility and allows the analysis of the quality of the report, which is why we suggest that it is the best tool for causality assessment of ADRs occurring in hospitals. Since the Naranjo algorithm was developed and validated to diagnose ADR occurring in randomized clinical trials and showed slight concordance between the judges, this tool is not the most consistent for the assessment of ADRs that affect non-critical patients in a secondary hospital. In addition, data demonstrate the need for the development of better quality tools, that include other criteria for the assessment of drug-related problems, such as effectiveness, safety, compliance, quality or quality deviation and medication errors.
